# A systematic evaluation of expression of HERV-W elements; influence of genomic context, viral structure and orientation

**DOI:** 10.1186/1471-2164-12-22

**Published:** 2011-01-12

**Authors:** Fang Li, Christoffer Nellåker, Robert H Yolken, Håkan Karlsson

**Affiliations:** 1 Department of Neuroscience, Karolinska Institutet, Stockholm, Sweden; 2 MRC Functional Genetics Unit, Department of Physiology, Anatomy and Genetics, University of Oxford, UK; 3 Stanley Division of Developmental Neurovirology, Johns Hopkins University School of Medicine, Baltimore, MD, USA

## Abstract

**Background:**

One member of the W family of human endogenous retroviruses (HERV) appears to have been functionally adopted by the human host. Nevertheless, a highly diversified and regulated transcription from a range of HERV-W elements has been observed in human tissues and cells. Aberrant expression of members of this family has also been associated with human disease such as multiple sclerosis (MS) and schizophrenia. It is not known whether this broad expression of HERV-W elements represents transcriptional leakage or specific transcription initiated from the retroviral promoter in the long terminal repeat (LTR) region. Therefore, potential influences of genomic context, structure and orientation on the expression levels of individual HERV-W elements in normal human tissues were systematically investigated.

**Results:**

Whereas intronic HERV-W elements with a pseudogene structure exhibited a strong anti-sense orientation bias, intronic elements with a proviral structure and solo LTRs did not. Although a highly variable expression across tissues and elements was observed, systematic effects of context, structure and orientation were also observed. Elements located in intronic regions appeared to be expressed at higher levels than elements located in intergenic regions. Intronic elements with proviral structures were expressed at higher levels than those elements bearing hallmarks of processed pseudogenes or solo LTRs. Relative to their corresponding genes, intronic elements integrated on the sense strand appeared to be transcribed at higher levels than those integrated on the anti-sense strand. Moreover, the expression of proviral elements appeared to be independent from that of their corresponding genes.

**Conclusions:**

Intronic HERV-W provirus integrations on the sense strand appear to have elicited a weaker negative selection than pseudogene integrations of transcripts from such elements. Our current findings suggest that the previously observed diversified and tissue-specific expression of elements in the HERV-W family is the result of both directed transcription (involving both the LTR and internal sequence) and leaky transcription of HERV-W elements in normal human tissues.

## Background

Recent analyses of the transcriptional landscape of human cells and tissues indicate that far more than the 2% of human genome that encodes proteins exhibit tissue-specific and regulated transcription resulting in a large number of different RNA species without apparent coding capacity [1]. Such analyses, however, focused on the non-repetitive regions and consequently little is known on the extent of transcription in repetitive regions of the human genome.

Five to eight percent of the human genome consists of retroviral sequences acquired during evolution [2]. These sequences can be grouped into at least 31 families of human endogenous retroviruses (HERV) [3]. Although expression of different HERV families has been associated to a range of human diseases and pathological conditions, very little is known of their basal expression, regulation and potential functional roles. A prominent exception, however, is the *ERVWE1 *locus on chromosome 7q. This locus harbors a member of the HERV-W family with an open reading frame in the *env *gene that encodes a protein denoted syncytin [4]. This protein is highly expressed in the syncytiotrophoblast layer of the human placenta and appears to have been functionally adopted by the human host for fusion of trophoblast cells and thus contributing to the formation of the syncytiotrophoblast layer [5].

**Figure 1:**
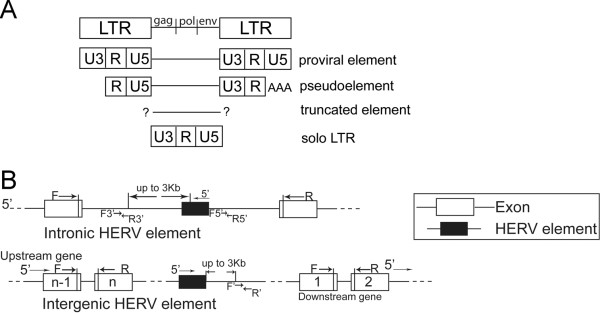
**Schematic structure of HERV-W elements and assay designs**. Schematic illustration of the structures of provirus, pseudoelement, truncated element and solo LTR (A). Strategy for design of assays specific for intronic and intergenic HERV-W elements and their corresponding genes. F3'/R3' and F5'/R5' denote forward and reverse primers in intronic regions 3' and 5' of HERV-W elements, respectively (B).

In addition to *ERVWE1*, the human genome harbors hundreds of elements in the HERV-W family. The prototypical HERV-W provirus consists of an internal sequence with the *gag*, *pol *and *env *genes flanked by identical 5' and 3' long terminal repeats (LTRs). In addition to such proviral elements (e.g. *ERVWE1*), elements that bear the hallmarks of processed pseudogenes (denoted pseudoelements) and elements that cannot be classified into either of these categories (denoted truncated elements) are present in the human genome [6,7]. Moreover, a large number of solo LTRs are present, probably remnants of homologous recombination events between the two LTRs of proviral elements, Figure 1A. Since the U3 region of the LTR typically contains the binding motifs necessary for transcriptional initiation, only proviral elements have been considered transcriptionally active [6,7]. Previous experimental studies have reported that cloned HERV-W LTRs can indeed induce expression of a reporter gene in human cells [8]. Studies of the proviral element in *ERVWE1 *locus also indicate promoter activity in the 5'-LTR as well as strong enhancer activity in flanking DNA conferring the specific and high level expression in syncytiotrophoblasts [9]. We recently profiled transcripts containing HERV-W *gag *sequences using high-resolution melting temperature analysis. While a highly diversified expression across both individuals and tissues was evident, tissue-specific profiles were also observed [10]. Mapping of HERV-W transcripts indicate that a range of both intronic and intergenic genomic regions harboring HERV-W elements are indeed transcribed in human cells and tissues. However, such transcripts mapped, not only to proviral elements, but also to pseudoelements and truncated elements [11-13], i.e. elements lacking the regulatory motifs in the U3 region of the 5'-LTR.

These observations raise questions regarding the mechanisms responsible for generating such transcripts. Do they constitute products of leaky transcription of adjacent genes, unprocessed pre-mRNAs or are they independently initiated from the retroviral LTR (or other promoters)? The purpose of the present study was therefore to systematically determine the influence of the genomic context, structure and orientation on the expression of HERV-W elements. Understanding the transcriptional regulation of HERV-W elements will contribute to a better understanding of some of the least studied regions of the human genome and can shed light on mechanisms underlying the associations between expression of HERV-W elements and complex human diseases, such as MS [14,15] and schizophrenia [16,17].

## Results

### Distribution of HERV-W elements in the human genome

Approximately 28% of genomic loci with HERV-W elements identified here were observed within introns of annotated genes. No significant difference in this distribution was observed between the three categories investigated, i.e proviral elements, solo LTRs and pseudoelements. As can be seen from Table 1 the majority of intronic elements are integrated on the anti-sense strand, i.e. in the opposite orientation to the surrounding gene. As has been previously reported [18], the proportion of anti-sense integrated HERV-W elements was significantly larger than that expected by chance (i.e. 50% on each strand). The proportion of proviruses integrated on the sense strand was similar to that of solo LTRs. Surprisingly, the proportion of proviruses and solo LTRs integrated on the sense-strand did not differ from that expected by chance. Only the proportion of intronic pseudoelements integrated on the sense strand was significantly lower than that expected by chance.

**Table 1:** Distribution of HERV-W elements.

		Proviruses	Solo LTRs		Pseudoelements			
Intergenic		37	110		66			

Intronic		10	43		29			

p-value			0.508 ^a^					

								

	**Total^1^**	Expected*	**Pro**	**Solo**	**Total^2^**	Expected*	**Pse**	Expected*

Sense	21	43	3	16	19	28	2	14

Anti-sense	65	43	7	31	38	29	27	15

p-value	0.0005^a^		1.000^b^		0.087^a^		0.0008^b^	

Distribution of three categories of HERV-W elements in genic/intergenic regions and distribution of intronic HERV-W elements on sense and anti-sense strands, respectively. ^1^Total number of intronic HERV-W elements, ^2 ^indicates number of proviruses and solo LTRs. * indicate expected number based on random integration (i.e. 50% on each strand). Pro, solo and pse indicate proviruses, solo LTRs and pseudoelements, respectively. ^a^Chi-square test, ^b^Fisher's exact test. P-values <0.05 were considered significant.

### Expression levels of intronic and intergenic HERV-W elements

**Figure 2:**
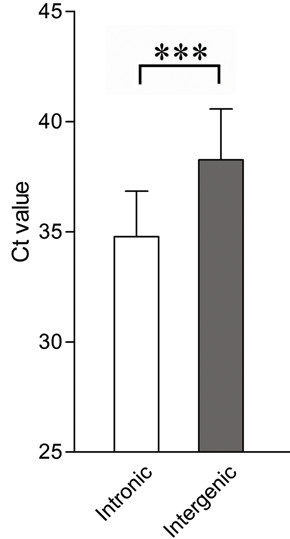
**Comparison of average expression levels between intronic (n = 14) and intergenic (n = 10) HERV-W elements in eight human tissues**. Transcript levels were determined by real-time PCR for each element using assays as described in material and methods and reported as Ct-values. Note that low Ct-values indicate high expression. Bars indicate means and error bars indicate standard deviations (SD). Statistical significance is indicated by ***= p < 0.001.

To investigate if the genomic context of HERV-W elements determines their expression levels, we assayed the levels of transcripts from 14 HERV-W loci located in introns of genes (i.e. intronic elements) and from 10 HERV-W loci located outside of annotated genes (i.e. intergenic elements, as illustrated in Figure 1B) in a commercially available human tissue cDNA panel. For this purpose we used real-time PCR assays, often directed at the most proximal non-repetitive region 3' of each element as detailed in the methods section. Overall, the normal human tissues contained higher levels (i.e. lower Ct-values) of transcripts harboring sequences 3' of intronic HERV elements than 3' of intergenic elements (Figure 2). This was a consistent finding across all tissues (additional file 1: fig. S1A). For reference, we also assayed expression levels of transcripts encoding syncytin in the individual tissues. Transcripts 3' of some intergenic elements could not be detected in some of the tissues. These were excluded from the calculations of mean expression levels presented in additional file 1: fig. S1A. It should be noted that individual elements exhibited substantial expression levels in some tissues but not in others. A large variation in the levels of expression of intronic and intergenic elements was observed across both elements and tissues (additional file 2).

### Expression levels of intronic elements, the roles of the U3 region and internal sequence

**Figure 3:**
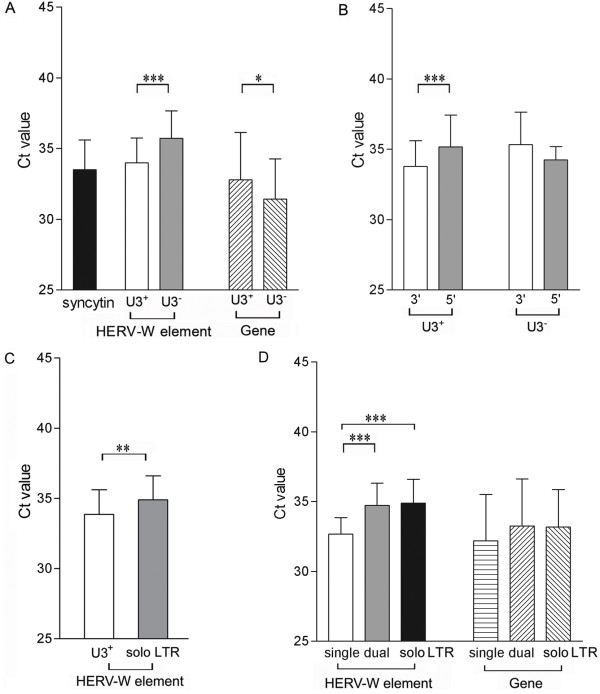
**Average levels (Ct-values, SD indicated by error bars) of transcripts 3' of intronic HERV-W elements and their corresponding genes as determined by real-time PCR**. Comparison of expression levels between proviral (n = 7) and pseudoelements (n = 7), as well as comparison of expression levels between the genes into which these elements are integrated. Expression levels of transcripts encoding syncytin is shown as a reference (A). Levels of transcripts 5' and 3' of proviral elements (n = 3) or pseudoelements (n = 3) (B). Levels of transcripts 3' of proviral elements (n = 7) and solo LTRs (n = 6) (C). Levels of transcripts 3' of proviral HERV-W elements with a single (n = 3), dual LTRs (n = 4) and 3' of solo LTRs (n = 6). Expression levels of the genes into which these elements are integrated are also indicated (D). Statistical significance is indicated by *= p < 0.05, **= p < 0.01, ***= p < 0.001.

We next investigated if the expression levels of the intronic HERV-W elements were influenced by their structure. Therefore, the levels of expression 3' of proviral elements were contrasted with those 3' of pseudoelements in the human tissues. The mean expression levels of transcripts encoding syncytin in normal human tissues were included as reference. Overall, transcripts from proviral (U3^+^) elements were detected at levels comparable to those encoding syncytin and at higher levels than those from pseudoelements (U3^-^, Figure 3A). This difference did not appear to be an effect of the degree of expression of the genes into which these elements were integrated since the genes harboring proviral elements investigated here, in fact, displayed lower levels of spliced mRNAs than the genes harboring pseudoelements (Figure 3A). These were consistent findings across all tissues investigated (additional file 3).

The findings of such systematic differences in expression levels suggest transcriptional initiation in the HERV-W LTR. To further investigate this issue, we randomly selected three elements from each of the categories of intronic proviral-and pseudoelements. These included elements integrated both sense and anti-sense to their corresponding genes. Assays directed at the most proximal non-repetitive region 5' of each of these elements were designed and employed on the eight normal human tissues. According to our analyses, transcripts containing regions 3' of proviral HERV-W elements were more abundant than those containing regions 5' of these elements. However, levels of transcripts containing sequences 3' and 5' of pseudoelements did not differ (Figure 3B). A comparison of the individual elements, indicate that regions 3' of two pseudoelements were in fact expressed at significantly lower levels than their corresponding 5' regions (additional file 1: fig. S1B). These elements in *FOXP2 *and *NRCAM*, respectively were both integrated on the anti-sense strand. Taken together, these findings indicate functional promoter activity of the proviral LTR.

Next we compared levels of transcripts 3' of intronic proviral elements with those 3' of intronic solo LTRs. This comparison indicated that transcription 3' of proviral elements was significantly higher than that 3' of solo LTRs (Figure 3C). Again, this difference was consistently observed across all tissues (additional file 3). To determine whether the additional LTR or the internal sequence present in the proviral elements contributed to this difference in expression levels we subsequently compared levels of transcripts 3' of intronic elements that contained both 5'- and 3'-LTRs (dual) with those 3' of intronic elements lacking either the 3'- or the 5'- LTR (single) and with those 3' of solo LTRs (solo). We noted higher levels of transcripts 3' of proviral elements with single LTRs than 3' of proviral elements with dual LTRs or solo LTRs (Figure 3D). Spliced transcripts from corresponding genes were all detected at comparable levels (Figure 3D).

### Expression of intergenic elements

Among the 10 intergenic HERV-W elements investigated, expression levels did not differ between proviral and pseudoelements (additional file 4: fig. S4A). The U3-region of the LTRs of intergenic proviral elements did therefore not appear to influence expression and these elements may be expressed as a consequence of transcriptional leakage. We reasoned that intergenic elements can potentially be transcribed as a consequence of read-through transcription of upstream genes. The likelihood of such events to occur would be expected to be highest for elements located close to highly expressed genes. Surprisingly, our analyses revealed an inverse correlation between the levels of transcription of intergenic elements and their downstream genes (additional file 4: fig. S4B), while no correlation was observed between expression levels of intergenic elements and their upstream genes. Moreover, no evidence for correlations between expression levels of intergenic elements and their distance from either upstream or downstream genes was observed.

### Effect of orientation of HERV-W element on expression

**Figure 4:**
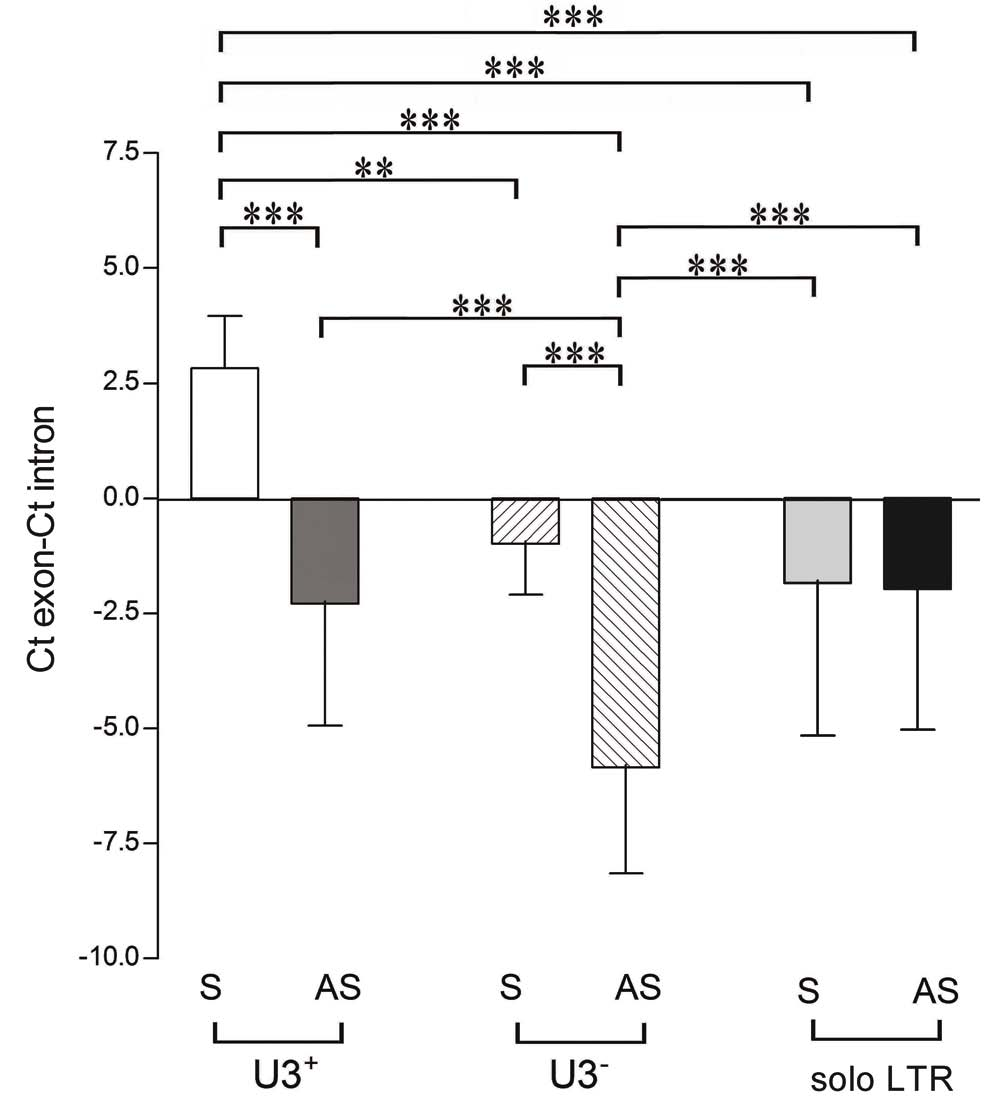
**Relative levels of transcripts 3' of different categories of HERV-W elements as determined by real-time PCR**. Levels of transcripts 3' of; intronic proviral elements, pseudoelements and solo LTRs are shown relative to expression-levels of their corresponding genes (i.e. ΔCt-values). Elements integrated on the sense (S) or anti-sense (AS) strand were analyzed separately. Statistical significance is indicated by **= p < 0.01, ***= p < 0.001.

To investigate if the orientation of HERV-W elements had any influence on their expression levels in relation to that of their corresponding genes, ΔCt's (Ct_gene_-Ct_element_) were calculated for each of the gene-element pairs. These ΔCt-values were subsequently compared across such pairs grouped according to structure (i.e. presence/absence of U3 region or solo LTR) and orientation (sense or anti-sense to the coding gene). As indicated by a one-way ANOVA, ΔCt-values between these six groups differed significantly (p < 0.0001, Figure 4). *Post-hoc *analyses indicated that proviral elements (U3^+^) in the sense orientation were expressed at levels exceeding those observed for the corresponding spliced protein-coding mRNA. These relative levels differed from the five other groups of elements investigated. Relative to their corresponding coding transcripts, proviral elements in the anti-sense orientation were expressed at higher relative levels than pseudoelements in the anti-sense orientation. Differences in the relative expression levels were also observed between pseudoelements in the sense and in the anti-sense orientations. Based on these observations it appeared as if intronic sense transcripts were present at approximately five cycles (i.e. ~32 times) higher levels than intronic anti-sense transcripts in the regions investigated. However, this finding was not consistent across all pairs since transcripts 3' of solo LTRs oriented sense and anti-sense were detected at similar relative levels (Figure 4).

### Dependent or independent transcription of intronic HERV-W elements

**Figure 5:**
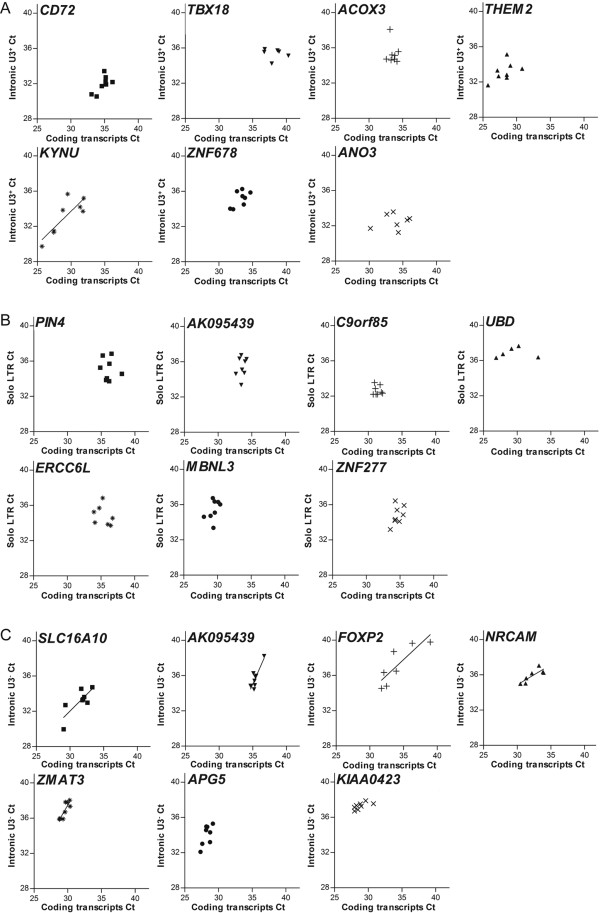
**Linear regression analyses of levels of transcripts 3' of intronic HERV-W elements and the genes into which they are integrated**. Linear regression analyses of levels of transcripts 3' of proviral HERV-W elements (A), solo LTRs (B), pseudoelements (C) and their corresponding genes.

We next tried to determine if the expression of intronic HERV-W elements was dependent or independent of that of the coding gene into which the elements were integrated. For that purpose linear regression analyses were performed to examine whether the levels of transcripts 3' of intronic HERV-W elements were determined by the levels of corresponding coding transcripts. Indeed, in the different tissues investigated, regression lines with slopes significantly deviating from zero were observed for five out of the seven pseudoelements investigated. In contrast, a significant correlation was observed for only one of the seven proviruses, and for none of the five solo LTRs, investigated (Figure 5). For two of the solo LTRs, linear regression analyses could not be performed due to insufficient expression levels of either the LTR or the gene, resulting in too few data points for analysis. In conclusion, the levels of transcripts 3' of most pseudoelements, but not 3' of proviral elements or solo LTRs, appeared depend on the level of transcription of the gene into which they are integrated.

### Regulated transcription of HERV-W elements

**Figure 6:**
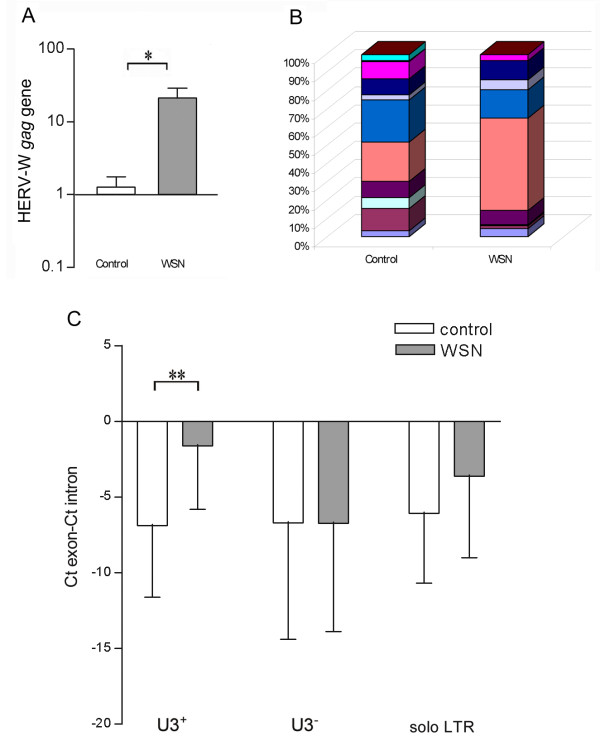
**Transcription of HERV-W elements in response to virus infection**. Relative levels of transcripts containing HERV-W *gag *sequence in uninfected human dermal fibroblasts (control) and in such cells 24 hours after infection with 0.5 MOI of influenza A virus (WSN, A). Frequency distributions of Tm categories of transcripts containing HERV-W *gag *elements in uninfected (control) and infected (WSN) human dermal fibroblasts (B). Relative levels of transcripts 3' of HERV-W proviral elements (U3^+^) (n = 7), pseudoelements (U3^-^) (n = 7) and solo LTRs (n = 4) in control and in influenza A/WSN/33 virus infected cells. Levels of all transcripts were normalized to expression-levels of their corresponding genes by pair-wise subtractions of the Ct-values obtained for assays in the flanking exons with those obtained from assays 3' of the intronic element (C). Statistical significance is indicated by *= p < 0.05, **= p < 0.01.

Previous studies from our laboratory indicated transcriptional activation of HERV-W elements, including *ERVWE1*, following influenza A/WSN/33 virus infection of different human cell-lines of non-placental origin [10]. To investigate potential quantitative and qualitative changes in HERV-W expression following influenza A virus infection in primary human cells, fibroblast cultures from a single individual were studied. As compared to uninfected control cultures, virus-infected cells contained ~20-fold higher levels of transcripts containing HERV-W *gag *sequences, Figure 6A. The number of different HERV-W loci expressed during base-line (control) conditions in these cells is evident from a high-resolution Tm-analysis [19] of the amplicons generated by the assay directed at HERV-W *gag*, Figure 6B. The change in the profile of such transcripts by the virus infection is also illustrated by the Tm-profile in infected cultures (WSN), Figure 6B. To further explore the potential role of the HERV-W LTR in this transcriptional activation of HERV-W elements, we investigated the levels of transcripts 3' of HERV-W elements relative to their corresponding genes in these cultures. ΔCt's (Ct_gene_-Ct_element_) were calculated as described above. As can be seen from Figure 6C, proviral elements (U3^+^) appeared to display significantly elevated transcriptional activities following influenza A/WSN/33 infection relative to their corresponding coding transcripts as indicated by the significant increase in average ΔCt-values. A similar but non-significant finding was also observed for solo LTRs. In contrast, no relative change was observed for transcripts 3' of pseudoelements (U3^-^) by comparing uninfected control and infected cells.

## Discussion

As for other HERV-families, the majority of surviving elements in the W family are located outside of protein-coding genes [18]. The percentage of intronic elements in this family (28%) is comparable to the percentage of bases spanned by human genes [20,21] suggesting that these integrations were not biased with regard to genic or intergenic regions. It should be noted that the unbiased integration also applies to pseudoelements which presumably have integrated by L1-mediated retrotransposition of proviral transcripts [6,7] that differ from the original proviral integrations. Throughout the human genome, solo LTRs outnumbered proviral elements 3.2:1 suggesting that homologous recombination has occurred frequently during human evolution. The distribution of solo LTRs to genic and intergenic regions was comparable to that of proviral elements suggesting that such homologous recombinations were also neutral with regard to genic/intergenic regions. As has been previously reported [18], intronic elements exhibit a strong anti-sense bias. The proportions of proviral elements and solo LTRs integrated on the anti-sense strand did not differ suggesting that homologous recombinations were equally common on the sense and on the anti-sense strands in genic regions. In fact, the total proportion of proviral elements and solo LTRs on the sense strand did not differ significantly from that expected by chance. It seems, rather, that it is the small proportion of intronic pseudoelements that have survived on the sense strand that accounts for the overall anti-sense bias of intronic HERV-W elements. This latter observation indicates that pseudogene integrations have elicited stronger negative selection than proviral integrations on the sense strand of coding regions. Alternatively, this finding indicates strand-preference of L1-mediated integrations.

In the present study we found that the genomic context, structure and orientation of elements in the HERV-W family appear to influence their expression. While systematic effects of these features were observed throughout a range of normal human tissues, it should also be noted that a considerable degree of variation in transcripts levels was observed across individual tissues and elements. From a methodological point, it should here be noted that the variation, evident in additional file 2 strongly argues against any significant contribution to the results demonstrated here by contaminating genomic DNA. Moreover, the absence of detectable target transcripts by some of the assays in some of the tissues offers further support for this argument. The current findings are thus in general agreement with previous reports of wide-spread expression of HERV-W elements in human cells and tissues [11-13]. The quantitative differences observed here for individual elements across human tissues are also in line with previous studies of a small number of individual HERV-W elements in human cell-lines [12] and of the collective expression level of HERV-W elements in human tissues [16,22,23]. The current observations also support previous sequencing and mapping studies detecting transcription from HERV-W elements located in both intronic and intergenic regions [11-13].

Based on the current data, intronic elements appear to be, in general, expressed at higher levels than their intergenic counterparts, regardless of the structure of their LTR. In their study of human-specific HERV-K promoter activities, Buzdin and coworkers, also observed a major influence of genomic context on the activity of HERV-K LTRs. A larger proportion of LTRs in gene-rich regions appeared transcriptionally active as compared to LTRs in gene-poor regions [24]. Taken together, these finding thus appear to be in general agreement with a recent study by van Bakel and coworkers [25]. Based on data from transcriptome-sequencing of human tissues, these authors concluded that the abundance of intergenic transcripts was low compared to transcripts from known genes. Of the intronic HERV-W elements investigated here, those with a proviral structure were expressed at higher levels than those with a pseudoelement structure. While this might have been a consequence of higher transcriptional activities of genes with proviral integrations, our observations indicate the reverse to be true. Spliced transcripts from genes with proviral element integrations were in fact expressed at significantly lower levels than those from genes with pseudoelement integrations in all tissues investigated. These systematic findings suggest functionality of the U3-region of the proviral LTR in initiating and regulating transcription of proviral HERV-W elements *in vivo*. To avoid issues regarding specificity and sensitivity, we here assayed transcription harboring non-repetitive regions proximal to the 3' end of the HERV-W elements investigated. Genomic distances were verified by PCRs on human genomic DNA. Clearly distinguishable amplicons of expected sizes were, however, generated only after careful optimization illustrating the difficulties involved when using primers in repetitive elements. By the approach taken here, transcriptional initiation outside of the LTR cannot be distinguished from LTR-initiated transcription. Moreover termination of transcription 5' of the assayed region would lead to underestimations of transcription levels. In only 3 out of 22 cases were we unable to "link" elements with their corresponding non-repetitive 3'-regions at the level of transcription in the samples investigated. Distance of the assayed region from the site of oligo-d(T) initiated reverse transcription can also confound results in this type of study. By comparing the transcriptional activity 5' with that 3' of six intronic HERV-W loci in eight tissues we observed significantly higher expression level 3' of proviral elements but not 3' of pseudoelements. This finding further support transcription originating in the U3 region of the LTR which is expected to lead to higher transcript-levels from regions 3' than 5' of the promoter. Expression levels of the two regions would presumably be more uniform if our assays detected the same un-spliced pre-mRNA of coding genes, as for the pseudoelements investigated. We even observed lower transcript levels 3' than 5' of the pseudoelements in the anti-sense orientation which may be due to a longer distance from the site-of-initiation of reverse transcription. These findings in normal tissues are thus in agreement with previous studies of the *ERVWE1 *locus in placental cells and a more recent study of intergenic HERV-W loci in testicular tumors where transcription of intergenic proviral elements appeared to be initiated in the U3 region of the LTR [26]. Functionality of the U3 region of the HERV-W LTR is further supported by the observations that also solitary LTRs appeared to have retained their potential to initiate transcriptional activity. The finding that intronic transcripts of proviral elements, particularly those with only one intact LTR, were detected at significantly higher levels than those of solo LTRs, however indicates that the internal sequence (i.e. *gag*, *pol *and/or *env*) also has some impact on the levels of transcripts generated. A similar observation was also observed among elements in the HERV-K family suggesting that regulatory elements are present in the internal sequence [24]. Noticeably, intronic proviral elements with a single LTR displayed higher levels of transcription than those containing dual LTRs, potentially indicating transcriptional termination in the 3' LTR. Such termination would thus lead to an underestimation of the transcriptional activities of proviral integrations by our current analytical approach.

Regardless of U3 region, the orientation of intronic HERV-W elements appears to be an important determinant of their level of expression with elements on the sense strand being expressed at higher relative levels than those on the anti-sense strand. That this does not apply to solo LTRs, further supports the conclusion that the internal sequences present in both proviral elements and pseudoelements can modify the level of transcription.

According to our linear regression analyses, transcription of intronic proviral elements and solo LTRs, in general, appeared to be independent of their corresponding genes. Expression of most, but not all, intronic elements lacking the U3 region on the other hand appeared to be dependent on expression of their corresponding genes.

Transcriptional regulation of HERV-W elements is further supported by our observations in primary human fibroblasts. The overall increase in relative levels of transcripts containing *gag *sequence following virus infection extends our previous findings in human cell-lines [12]. Our qualitative analyses of *gag *amplicons in such cultures serve to illustrate the large number of transcribed loci in these cells and the alterations in the proportions of transcripts from these following virus infection, as we previously observed in serum-starved cells [10]. Independent and regulated expression of individual HERV-W elements with a proviral structure was further supported by our findings in the fibroblast cultures. Only transcripts 3' of such elements increased beyond that of corresponding spliced transcripts. Taken together, these findings support the previously reported functionality of the U3 region of HERV-W LTRs [8,9] and indicate that transcripts containing intronic pseudoelements are more likely to represent unprocessed pre-mRNAs or leaky transcription of coding genes.

We observed an inverse correlation between expression of intergenic HERV-W elements and their corresponding downstream genes. These correlations appeared independent of viral structure and might therefore reflect general mechanisms such as structural changes in the chromatin [27] or methylation patterns of genomic regions upstream of transcribed genes [28,29].

In addition to the general influences of genomic context, structure and orientation noted above, other mechanisms are also likely to affect the level of transcription of individual HERV-W elements. For example, differences in methylation levels of the individual LTRs, as reported for LTRs in the HERV-E family [30] and for the 5'-LTR, in the proviral HERV-W element encoding syncytin [9,31] can contribute to variation across tissues. Gimenez and coworkers recently reported that while hypomethylation of the promoter domain of intergenic HERV elements appears to be a prerequisite for the increased expression observed in tumor as compared to normal tissue, the methylation status of the promoter does not predict expression levels [26]. Thus, sequence-differences between the different U3 regions affecting transcription factor binding motifs [8] are also likely to contribute to the variation in expression across individual elements observed here.

Earlier studies have reported that a number of endogenous proviral LTRs in the HERV-E, -I, -R and -H families have been co-opted during human evolution as alternative promoters of individual genes conferring additional transcriptional control [32,33]. More recent analyses of large-scale gene expression data indicate that thousands of cellular transcripts are in fact initiated at HERV LTRs suggesting that HERVs have significantly contributed to shaping the human transcriptome [34]. The tissue-specific distribution of HERV-W transcripts was illustrated in a recent high-resolution melting temperature analysis of expressed HERV-W *gag *sequences [10]. This approach indicated diversified and non-random expression-patterns of such sequences in human tissues. In fact, based on correlations between patterns of HERV-W *gag *expression, dendrograms could be constructed resembling those based on gene expression patterns. Based on the present findings we suggest that both LTR mediated and leaky transcription of the human genome contribute to such tissue-specific patterns of HERV-W transcripts. Thus, the aberrant HERV-W expression associated with complex human diseases such as MS [14,15] and schizophrenia [16,17] may be a reflection of cellular events acting on promoters of genes harboring HERV-W elements or on proviral LTRs. Current *in vivo *findings support previous findings *in vitro *[8] that the large numbers of solitary LTRs in the HERV-W family can also actively contribute promoter activity, as has been reported for solitary LTRs in HERV-K family [24]. If members of the HERV-W family, other than the proviral element in the *ERVWE1 *locus, contribute functional transcripts, however, remains to be established.

## Conclusions

We found a broad and variable expression of HERV-W elements in normal human tissues. Previously observed diversified and regulated expression of HERV-W elements appears to be due to both directed and leaky transcription of the human genome.

## Methods

### Identification of HERV-W elements in the human genome

HERV-W loci were identified by BLAT searches [35] of the human genome (March 2006 assembly) using the HERV17 internal sequence or the LTR17 sequence from Repbase Update [36]. Loci were defined as intronic if located within the boundaries of annotated RefSeq genes [37].

### Assay design

The assay for the *env *of the proviral element in the *ERWE1 *locus previously described [12] was used to determine levels of transcripts encoding syncytin. Based on genomic structure and orientation, 10 intergenic and 14 intronic loci and their corresponding genes were included in this study. In addition, 6 solitary intronic LTRs and their corresponding genes were included. Of the six solo LTRs, two were integrated into two distinct genes on opposite strands, whereas one was integrated into two versions of an annotated gene, Table 2. Specific quantitative assays for each of the targets were designed using Primer Express 2.0 (Applied Biosystems, Foster City CA, USA) and ordered from Invitrogen (Groningen, The Netherlands). In five cases, specific assays could be designed with at least one primer in the element. To avoid non-specific detection, assays for 25 elements were, however, designed in the most proximal non-repetitive region (according to RepeatMasker) 3' of each element. Distances between the last base of an element and the assayed region thus ranged between -3.2 and 3 kb (median 0.7 kb). By the same approach, assays 5' of six intronic elements were also designed. Distances from each of the elements were kept within 1.1 kb (median 0.6 kb).

**Table 2:** Primers used for assay design.

Target	LTR status	Polarity	Sequence	Position	Target	Polarity	Sequence	Position
Intronic HERV-W proviral element (3' region)					Gene harboring provirus			

Sense								
*CD72 *intron 1	3'	F	TGTCTCCAATTTTAACCCTATTTGC	chr9:35629741	*CD72 *exon 1~2	F	CTCTAGCTGATCGACTCACAAATACC	AK311283
		R	TTCCCCAGCCAGCATGTAA	chr9:35629792		R	TGAACATTCTCGTAGGTGATTTCC	
*TBX18 *intron 7	5'&3'	F	CCATTCCATGTTCCTCATTAAATCT	chr6:85477157	*TBX18 *exon 7~8	F	CCCAAGCAAGAATACAGGGACTT	BC040697
		R	AGGCAATCTTACCACCCAGAAG	chr6:85477208		R	GGTTTCCCTTGAGACCAAATTG	

Antisense								
*ACOX3 *intron 1	5'&3'	F	AGACGCTTGGAGCAGTTTCTG	chr4:8480630	*ACOX3 *exon 1~2	F	CTGTGGAGTGTGTGGGCTCTT	AK225080
		R	TCGGAGGACTTTACACATTGAACTAG	chr4:8480744		R	CAGAGCTGTGTCGCCTCCTT	
*KYNU *intron 2	5'&3'	F	AGTGGGAATACTGACGCTCTTACC	chr2:143372701	*KYNU *exon 2~3	F	ACAGGATCTGCCTCCAGTTGA	U57721
		R	GCCAAGAACCCAAGGTCAGA	chr2:143372792		R	TGGCCCACTTATCTAGTTCTTCTTC	
*THEM2 *intron 1	5'	F	TGAGATACCAGAGATCACCAAACTCTT	chr6:24787905	*THEM2 *exon 1~2	F	GGGAGGTGATAAAGGCCATGA	BC000894
		R	TGAAGTTTTATAAGGGTTAGGACAATCTT	chr6:24788017		R	GGAGCAGCAGAGACAAGAGTAATCT	
*ZNF678 *intron 1	5'&3'	F	GGAAATGAACATAGTCTTACTGTCAGGTA	chr1:225878139	*ZNF678 *exon 1~2	F	TGCAGGTACTGGGAGTTACATAGC	BC042500
		R	CATAGAGTCCAGAAATGTGTCTATCCA	chr1:225878254		R	TGCCAGTAGTCCCGTTTTCC	
*ANO3 *intron 14	3'	F	AAAGGATTGTGTAAGAGAAGCTGATAAGA	chr11:26568090	*ANO3 *exon 14~15	F	TTGTTCGATAATGGAGGGACAGT	BC172396
		R	GCAGAGATGTTATCCCTGTTGGT	chr11:26568167		R	CGATAAGGTCCCAAGTATAGGTCAGT	

Intronic HERV-W proviral element (5' region)								

*CD72 *intron 1		F	GCAGAGGTCTACTCACACCATAGC	chr9:35633414				
		R	GGCAATCAGGAGGCCTAAA	chr9:35633462				
*TBX18 *intron 7		F	GGGCTACAGTGATCCATTTATAAGG	chr6:85488459				
		R	AAACTTTATCCAAACATTCCCTATGTTAG	chr6:85488532				
*ANO3 *intron 14		F	TCTCCTTCACCACGAGAGTTTG	chr11:26576040				
		R	TGCTACTCGTCCAGAGCTTACAGA	chr11:26576091				

Intronic HERV-W pseudoelement (3' region)					Gene harboring pseudoelement			

Sense								
*SLC16A10 *intron 1		F	TGGCAGTGCTCGTTGAATGT	chr6:111566680	*SLC16A10 *exon 1~2	F	GGCTCCAAAGACGATGACAAG	BC034031
		R	ACATAAGCCCCAAAACTGTCAGA	chr6:111566728		R	AAATCATCCCCATGGAGAGAGA	
*AK024261 *intron 1		F	GGCCCACCTCCAGCAAA	chr2:218160716	*AK024261 *exon 1~2	F	CAGCAGATGATGGTGATGGAA	AK024261
		R	TGGGTGTGATCAGGTCATGGT	chr2:218160778		R	CATCCCTCGGACTCAGTTTCTC	

Antisense								
*FOXP2 *intron 3		F	CAAGAGGACATCTCTAAATCAAAGACTGT	chr7:113805180	*FOXP2 *exon 3~4	F	GCGAGGTTAATTCGAAAGTCTTGTGGAA	AF454830
		R	GAGCATGCCAGGCAAAGC	chr7:113805235		R	TTGCTTATTGTCTCTGTCGCAGAT	
*APG5 *intron 6		F	TCAAATCACACAATGCTCCAATG	chr6:106791850	*APG5 *exon 6~7	F	ATCCCCTTTAGAATATATCAGACAACGA	BC093011
		R	TTAGCAGTTGCATATGAGATCTTTCA	chr6:106791905		R	CCTAGTGTGTGCAACTGTCCATCT	
*KIAA0423 *intron7		F	GACATGAATATTTTTTGTGGACATAACTC	chr14:44557550	*KIAA0423 *exon 7~8	F	CCAACAAGACTTTCTTCTGCAAAG	AB007883
		R	GCCAAACAGAACAGTACTAAACTATATTATAGACT	chr14:44557636		R	TTTGCTGTGGATTTGATGATGAC	
*NRCAM *intron 2		F	AAGTCTGGGTTCCAGGTTCATC	chr7:107772873	*NRCAM *exon 2~3	F	GCTGGTCTTAGCTGTTGGCATT	BC115736
		R	CTGAGGAACCTTTCTGAGTTGAGTAA	chr7:107772929		R	CGTCTTCTTAGCATTGCTCTTCAG	
*ZMAT3 *intron 2		F	GGATTCCCAGGTACGGTGAAA	chr3:180258291	*ZMAT3 *exon 2~3	F	CTCTGCAATGTCACCTTGAACTCT	BC002896
		R	CCTAGAAAGCTACTTCCGCTTCAG	chr3:180258381		R	GCTGCATAGTAATTTCGGAGTTTCT	

Intronic HERV-W pseudoelement (5' region)								

*SLC16A10 *intron 1		F	CAAGAGGACATCTCTAAATCAAAGACTGT	chr6:111558508				
		R	GAGCATGCCAGGCAAAGC	chr6:111558555				
*FOXP2 *intron 3		F	TCAAATCACACAATGCTCCAATG	chr7:113813845				
		R	TTAGCAGTTGCATATGAGATCTTTCA	chr7:113813901				
*NRCAM *intron 2		F	GACATGAATATTTTTTGTGGACATAACTC	chr7:107763923				
		R	GCCAAACAGAACAGTACTAAACTATATTATAGACT	chr7:107763973				

Intronic solitary LTR					Gene harboring solitary LTR			

Sense								
*ZNF 277 *intron 1		F	AGAACCCAGGTCAGAAAACAAGAG	chr7:111648615	*ZNF277 *exon 1~2	F	GGCTGTCGCCCGAATG	AF209198
		R	CAGTCCCTGGTGCCAAGAAG	chr7:111648735		R	ACAATCCTTACTGTCCCCATAACC	
*WDR72 *intron 14		F	AAACACTATCTGATTGCCTGACAGA	chr15:51704774	*WDR72 *exon 14~15	F	CCAGCTTGGGCCATTACCTT	BX53788
		R	CCAGGACCTTGTCTATGATGCA	chr15:51704828		R	GCAAAATTTGGCATCAGTAACCT	
chrXq13.1		F	CAGGTCCATTTAAAGCCCAAGT	chrX:71350691	PIN4 exon 3~4	F	CAGGTCCATTTAAAGCCCAAGT	AK12760
		R	CCTGAAAGTGAAGCCAAAAGGA	chrX:71350741		R	CCTGAAAGTGAAGCCAAAAGGA	
chrXq26.2		F	TGGGAGAAGCAATGTCCAATT	chrX:131380909	MBNL3 exon 1~2	F	TGGCCTGTTTTGATTCTCTAAAGG	AB077698
		R	TTTGTCAAGTCATGGCCGTTT	chrX:131380961		R	AGTGTGGAGGAGGGTGAAGGT	

Antisense								
*C9orf85 *intron 2		F	ACCCACCAATTCCGGATACA	chr9:73769452	*C9orf85 *exon 2~3	F	GAGTGGCGTGTAAAATACAGCAAA	BC052375
		R	TGGCCCAAGATTTCATTCCT	chr9:73769523		R	CATGGCCTGCACATTATGTGA	
					*UBD *exon 1~2	F	GATGGCTCCCAATGCTTCCT	AF123050
						R	TGGCATCAAAGGTCATTAAATCC	
chr6p22.1		F	GCCAGATAACCAGCTTCTCTTCA	chr6:29635237	*GABBR1 *exon 18~19	F	GGAGTCGAGTGCATTTGCTTTAT	BC051463
		R	GAGAAGAGGCTGGCATTGACA	chr6:29635286		R	TGGCATCAAAGGTCATTAAATCC	
chrXq13.1					*ERCC6L *exon 1~2	F	GGAAGCCGAGGCCTTGAG	BC111486
						R	TTAGTTGCTTCTTTGGCCTCTTTC	
chrXq26.2					*AK095439 *exon 2~3	F	ACTTCATTGAATGTCTTAGACGGAAA	*AK09543*
						R	TCACATATGCAGTTATCAAAAGAGCTT	

Intergenic HERV-W proviral element and adjacent genes					Intergenic HERV-W pseudoelement and adjacent genes			

*LRIG3 *(5')		F	TCGAGTAATTCTTTCATGGGTACCT	AY505340	*COBLL1 *(5')	F	CAGAGTTTGCTGACTGCAATCC	BC071588
		R	ATCTGATGGCTGTCCAAAGCTT			R	GACCTTCCATTCACAGATATTGTATTTG	
pro chr12q14,1		F	TGGTCAAGAGGAGTCCAGAGTTT	chr12:57530852	pse chr2q24,3	F	ACCTACACCAATTCAGCTGTTAAGG	chr2:165222305
		R	GCCCACCAATGCAATGCT	chr12:57530902		R	CCACAATGCTCCGAGTTCACT	chr2:165222360
*AK093124 *(3')		F	TGTCCCTTGCTACATACCTGCTT		*GRB14 *(3')	F	TGTGCTGCAGACAGGCGAAAA	
		R	TGCACTCAGATAAAGTGTCAATAAGAAG	AK093124		R	CAGATGTAAATGGAGAACAGCATAGC	BC053559
*GSTA4*			N/A		*GRK7 *(5')	F	TGCAGGCTCTTCTTGGCTAAG	AF439409
						R	GTTTCCTGGGATCATCAGAATTTT	
pro chr6p12,1		F	GACCTTTTTCCTGGGCATCA	chr6:52887125	pse chr3q23	F	GGGTCCAAGGCTGCTCCTA	chr3:143023903
		R	GGGTAGGTGAACATGCCAGAA	chr6:52887169		R	GGTAAGCCTCCTGCACTCTGA	chr3:143023960
*GSTA3 *(3')		F	GCGGAGACCGGCTAGACTTT		*ATP1B3*		N/A	
		R	CCGTCCATTGAAGTAGTGAAGCT	BC020619				
*AKR1B10 *(5')		F	TGCTGAGGAGATGGCAACCAT	B C008837	*CLSTN3 *(5')	F	GGGATGACTCAGCTCTCACCAT	BC039075
		R	TCCAAATGAGAGGATTGCAACAC			R	TCACACAGGACTGCCGATTC	
pro chr7q33		F	TGGAAGAAACAAATTGCCTGATC	chr7:133929406	pse chr12p13.31	F	CAGAAAGGTTTTGCCTAGTAGTGTTG	chr12:7230506
		R	AAATGCCCTTAGGCCAGATAGAT	chr7:133929455		R	TGTGCTGTACTTCTGCTTTTTAGAAAT	chr12:7230605
*BPGM *(3')		F	GGTGGCCCCTTTGCAGAT		*PEX5 *(3')	F	ATGCAGCAGATTGAGCAGTCA	
		R	TGGACATACTGATGGCTGAACTTC	BC01705		R	CAGTTCTCAGACAAGGCCAAGTC	AK301700
*TMEM26 *(5')		F	TGCCATCCTTGTTATATGGACTTG	CR749606	*INTS7 *(5')	F	CACTGCAGAGTAAATCTGGACAAGA	BC020523
		R	CAGGGCACACAACGTTCTGT			R	TCAACCCTTTGCTCCATCTCA	
pro chr10q21.2		F	TGAAACGTGTCATGTATCATCTCAA	chr10:62462501	pse chr1q32.3	F	TCTGATGAACTCTGAATAAACTCTTTCT	chr1:210095554
		R	GGTAACACCCGGCCATTTT	chr10:62462549		R	G	chr1:210095648
*RHOBTB1 *(3')		F	CCGCGGGAGCTTCCA		*LPGAT1 *(3')	F	GATTGCATGCCCTAATTATGGAA	
		R	CCATTTTCATAACTTAGTCAAGAATGCT	BC093035		R	CCCTGAGAGATCACCGAGAAAG	BC034621
*LRRK2 *(5')		F	GGTACACAAAAGCAGAAAGAGATACAA	chr7:133929455	*RNGTT *(5')	F	GCCACGGAGCTTCTTCCA	BC019954
		R	TTTGCACTTCATGTGGAAGATTG			R	CAAAAGTTTTCCTAATGCCTACAACA	
pro chr12q12		F	GGGAGCAGATGAGGAGCAAA	chr12:39072588	pse chr6q15	F	CATCTCCTTGGTGACAGGGTTT	chr6:89180050
		R	GGTGGACCTGGAAAATGGAA	chr12:39072637		R	TGGTCACTTGATTTATAAGAAACGTGTT	chr6:89180152
*CR749517 *(3')		F	TTTGACTTACTCGTTCACTGTGATCA		*CNR1 *(3')	F	CCACATAGGACTGGTTTTCAATTG	
		R	TGTGGCTGCTGTGCTGAAA	CR749517		R	ATGCCTCCACCAGTGATTGC	AK313908
							GGCCATCTAGGATCGACTTCAT	

Target, primer sequences and position of sequences used for assay design. The same orientation of HERV-W element or solitary LTR and genes harboring such elements is indicated by sense, whereas opposite orientation is indicated by anti-sense. For simplicity, intergenic HERV-W elements transcribed in the same orientation as their adjacent genes were selected. Genes located upstream and downstream of intergenic elements are labeled 5' and 3', respectively.

To verify genomic locations and to link HERV-W elements and their corresponding assayed 3'-regions at the transcript level, forward primers inside 22 elements were designed (additional file 5: table S1). Following optimization, these primers in combination with corresponding reverse primers from quantitative assays located less than 2 kb 3' of corresponding HERV-W elements generated amplicons of expected sizes from human genomic DNA (data not shown). Following optimization, amplicons of expected sizes were also generated by most of 22 pairs using cDNA templates sourced from primary human cells or cell-lines (JEG-3 or CCF-STTG1). However, one assay still generated several bands whereas 3 assays produced no detectable product. These latter reactions covered the longest distances, i.e. 1.2 to 1.7 kb (data not shown).

Primers in assays for coding mRNAs were designed to detect exons flanking the introns with integrated HERV-W elements. Assay designs are illustrated in Figure 1B, and the sequences and positions of primers used for real-time PCRs are given in Table 2.

### Tissues

A commercially available Human MTC panel consisting of oligo-d(T)-primed cDNA from spleen, thymus, prostate, testis, ovary, intestine, colon and peripheral leukocytes was purchased from Clontech (Mountain View, CA, USA). These tissue cDNA were diluted 1/10 to reach a concentration of 0.1 ng/μl and then used as templates.

### Cell culture and influenza A/WSN/33 virus infection in cells

Fibroblast cell cultures were established as previously described [10], The study was approved by the regional ethics committee (04-273/1; 2006/637-32). Cultures derived from one individual were inoculated with 0.5 multiples of infection of influenza A/WSN/33 virus, as previously described [12]. Experiments were terminated after 24 hours by removal of supernatants and addition of lysis buffer (Qiagen, Hilden, Germany).

### RNA isolation and reverse transcription

RNA extraction and reverse transcription was carried out as previously described [12] using reagents from Qiagen. 500 ng of total RNA from each sample was treated with 1 U of DNase I (Invitrogen, Paisley, UK) and subsequently used for first strand oligo-d(T) primed cDNA synthesis using Superscript II reagents (Invitrogen).

### PCR and data analysis

PCR reactions were run on a GeneAmp PCR System 9700 (Applied Biosystem, Palo Alto, CA, USA) using 1× TITANIUM™Taq DNA polymerase mix (Clontech Laboratories Inc., Mountain View, CA, USA) and 1 μl template from in 20 μl reactions. Amplicons were separated in 1% agarose gels, stained with GelRed (Gentaur, Brussels, Belgium) and visualized on a Chemi Doc system (Bio-Rad, Hercules, CA, USA).

Real-time PCR reactions were run on an ABI Prism 7000 sequence detection system (Applied Biosystems) using Platinum SYBR Green qPCR SuperMix UDG (Invitrogen) with 0.25 μM of forward and reverse primers, and 1 μl cDNA template in triplicate 25 μl reactions. All assays consistently generated amplicons with a single melting temperature (i.e. a single peak on the derivative melting curve) as determined during an obligate post-amplification melting analysis. Efficiencies for all assays were comparable as determined from standard curves generated from amplifications of serial dilutions of a human cDNA pool (average efficiencies were 0.95 and 0.96 for the assays of HERV-W elements and adjacent genes, respectively). For some experiments, levels of transcript encoding β-actin were used as an endogenous control and these were quantified using the forward (5'-ATCCTAAAAGCCACCCCACT-3') and reverse (5'-CTCAAGTTGGGGGACAAAAA-3') primers.

Quantitative and qualitative analyses of HERV-W *gag *expression were performed as previously described [10].

Threshold cycle (Ct) values from the exponential phase of PCR amplification plots for each target transcript were exported to Excel (Microsoft Corporation, Redmond, WA, USA) for further calculations of averages or Δ Ct-values. For some experiments, Ct values were normalized to that encoding β-actin. From these values, fold-differences in the levels of transcripts among groups were calculated according to the formula 2 ^-ΔΔCt ^[38]. The PRISM software (GraphPad 3.0, San Diego, CA, USA) was used for group-wise comparisons by Student's t-test, Chi-square, two-tail Fisher's exact test or one-way ANOVA followed by Bonferroni-corrected multiple comparison *post hoc *tests where appropriate. P-values < 0.05 were considered significant.

## Authors' contributions

FL designed the experiments, performed the analyses and drafted the manuscript. CN performed statistical analyses and revised the manuscript. RY revised the manuscript. HK conceived the study and revised the manuscript. All authors have read and approved the final manuscript.

## Supplementary Material

Additional file 1 **Average levels of transcripts 3' of intronic/intergenic HERV-W elements across human tissues**. Levels of transcripts (as indicated by average Ct-values) 3' of intronic (n = 14) and intergenic (n = 10) HERV-W elements across human tissues. Levels of transcripts encoding syncytin are also shown as a point-of-reference (A). Levels of transcripts 5' and 3' of individual elements (B). Transcript-levels of elements were determined by real-time PCR using assays described in materials and methods. Click here for file

Additional file 2 **Relative expression of HERV-W elements and their corresponding genes**. Relative levels of transcripts 3' of intronic (A), intergenic (B) HERV-W elements, spliced exons flanking intronic HERV-W elements (C) and spliced transcripts from genes located 3' or 5' of intergenic HERV-W elements (D) across tissues and elements/genes. Levels of transcripts encoding β-actin were here used as an endogenous control. Click here for file

Additional file 3 **Average levels of transcripts 3' of intronic HERV-W elements and their corresponding genes across individual tissues**. Levels of transcripts 3' of proviruses, pseudoelements and solitary LTRs (A). Levels of spliced transcripts using primers in exons flanking introns where such elements are integrated (B). Click here for file

Additional file 4 **Average levels of transcripts 3' of intergenic HERV-W elements and adjacent genes**. Average levels of transcripts 3' of intergenic proviral elements (U3^+^, n = 5) and pseudoelements (U3^-^, n = 5) (A). Linear regression analyses of levels of transcripts 3' of intergenic U3^+ ^and U3^- ^elements and corresponding downstream genes (B). Click here for file

Additional file 5 **Primers located inside HERV-W elements**. This file describes the targets, product sizes, sequences and positions of primers used to link HERV-W elements with their corresponding assayed 3'-regions. Click here for file
