# Supplementary figures and images for: A systematic evaluation of expression of HERV-W elements; influence of genomic context, viral structure and orientation
Source: BMC Genomics. 2011 Jan 12;12:22. doi: 10.1186/1471-2164-12-22 (PMC3031232; doi:10.1186/1471-2164-12-22)

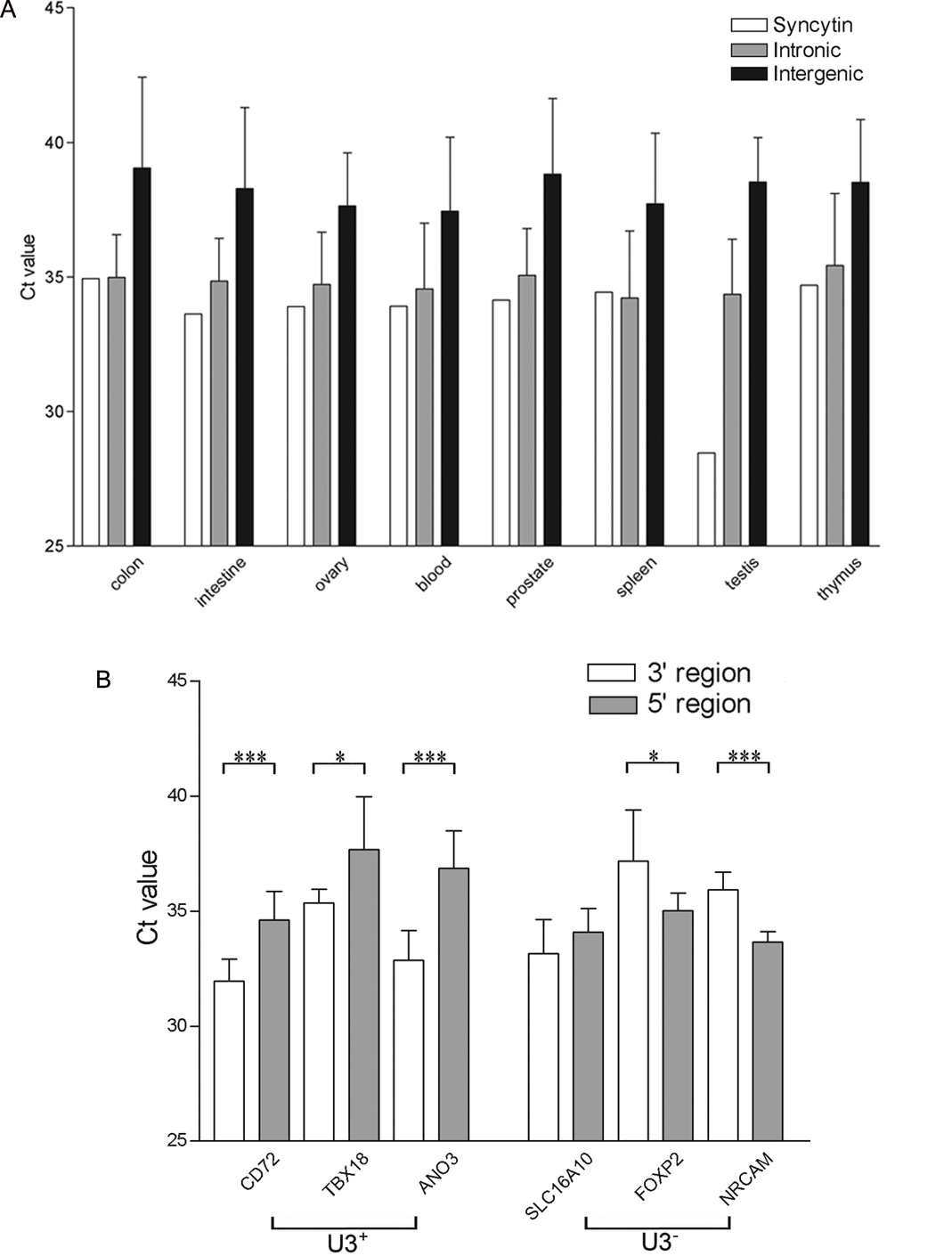

Supplement: Additional file 1 — Average levels of transcripts 3' of intronic/intergenic HERV-W elements across human tissues. Levels of transcripts (as indicated by average Ct-values) 3' of intronic (n = 14) and intergenic (n = 10) HERV-W elements across human tissues. Levels of transcripts encoding syncytin are also shown as a point-of-reference (A). Levels of transcripts 5' and 3' of individual elements (B). Transcript-levels of elements were determined by real-time PCR using assays described in materials and methods. [file 1471-2164-12-22-S1.TIFF]

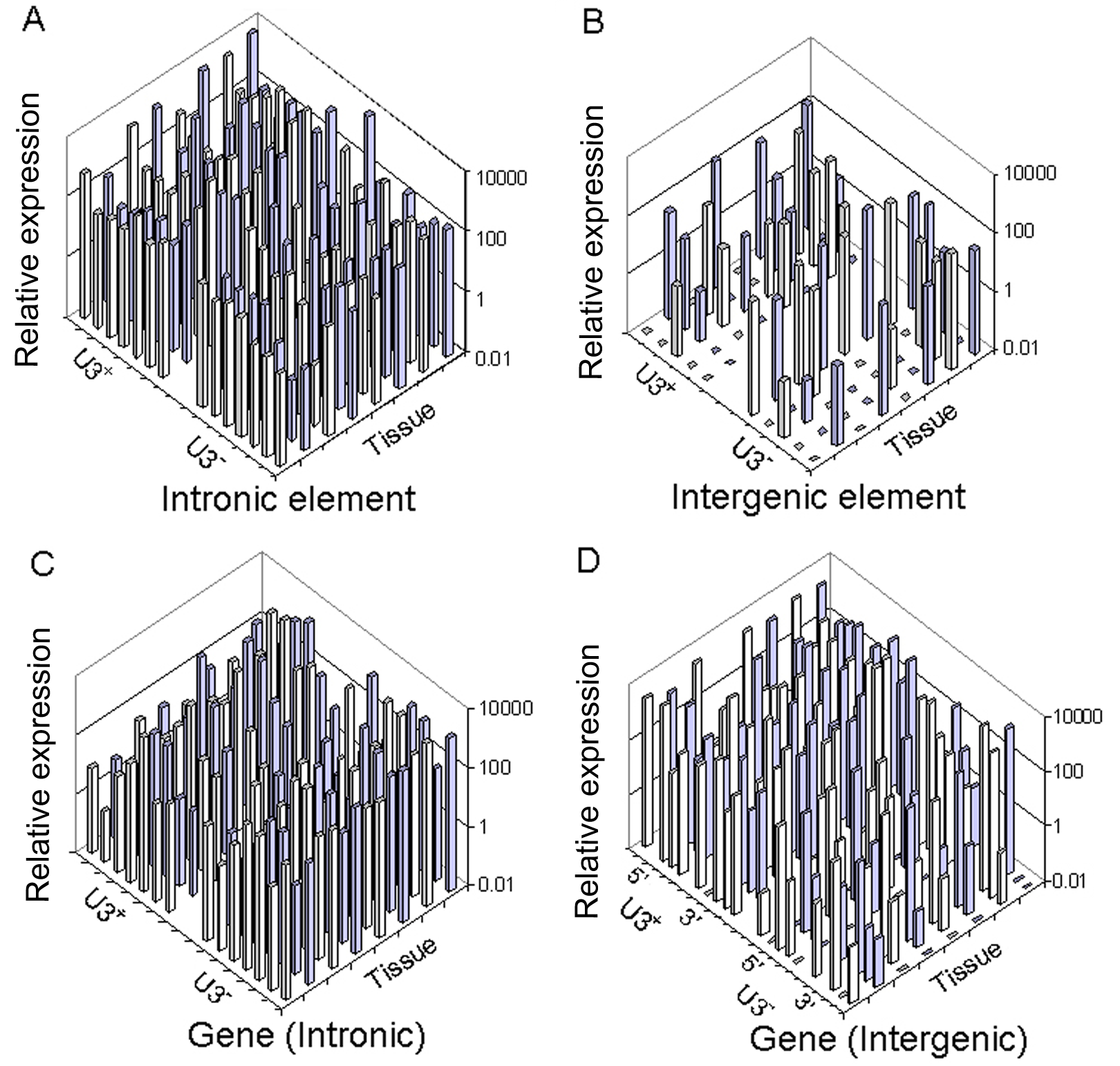

Supplement: Additional file 2 — Relative expression of HERV-W elements and their corresponding genes. Relative levels of transcripts 3' of intronic (A), intergenic (B) HERV-W elements, spliced exons flanking intronic HERV-W elements (C) and spliced transcripts from genes located 3' or 5' of intergenic HERV-W elements (D) across tissues and elements/genes. Levels of transcripts encoding β-actin were here used as an endogenous control. [file 1471-2164-12-22-S2.TIFF]

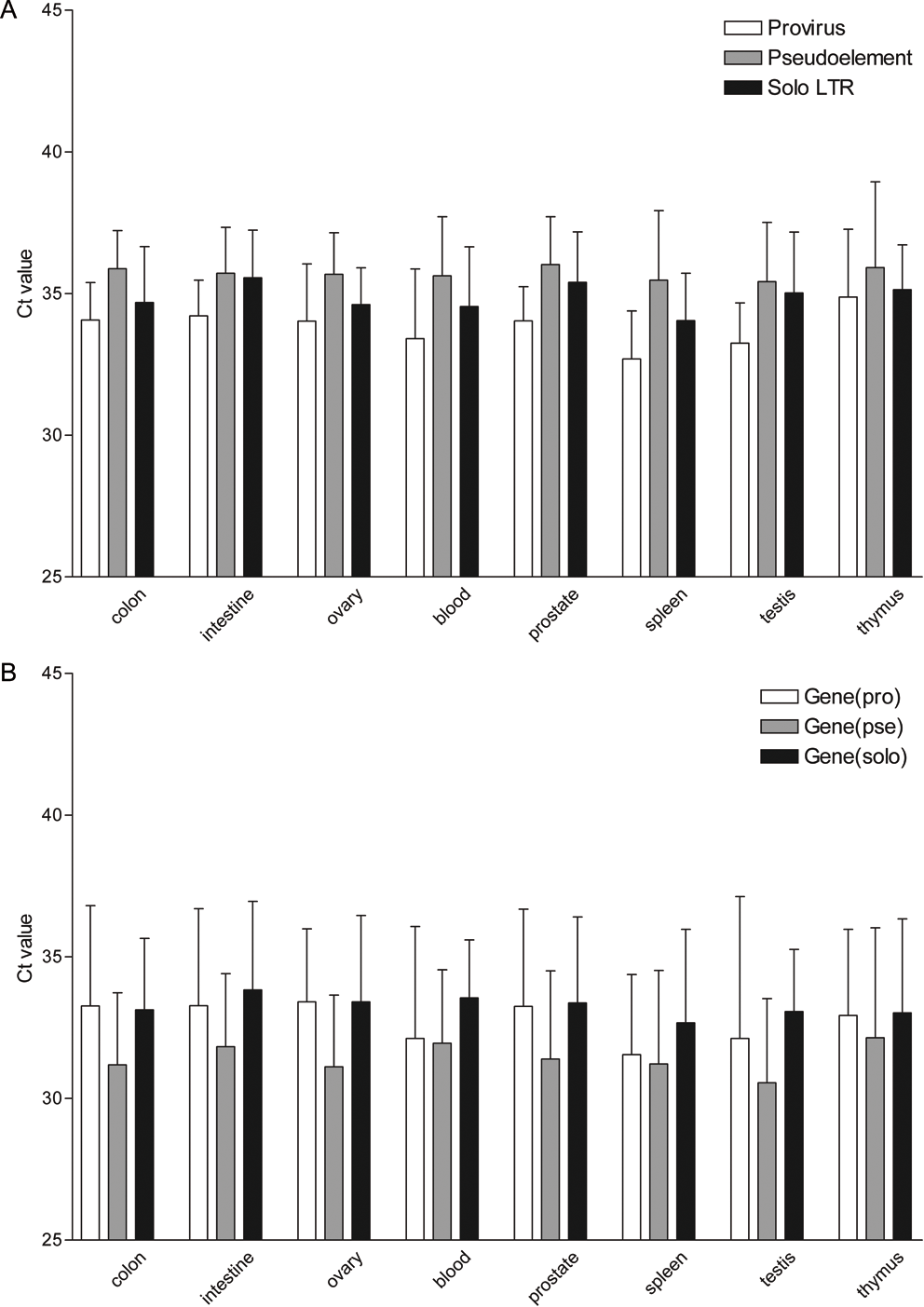

Supplement: Additional file 3 — Average levels of transcripts 3' of intronic HERV-W elements and their corresponding genes across individual tissues. Levels of transcripts 3' of proviruses, pseudoelements and solitary LTRs (A). Levels of spliced transcripts using primers in exons flanking introns where such elements are integrated (B). [file 1471-2164-12-22-S3.TIFF]

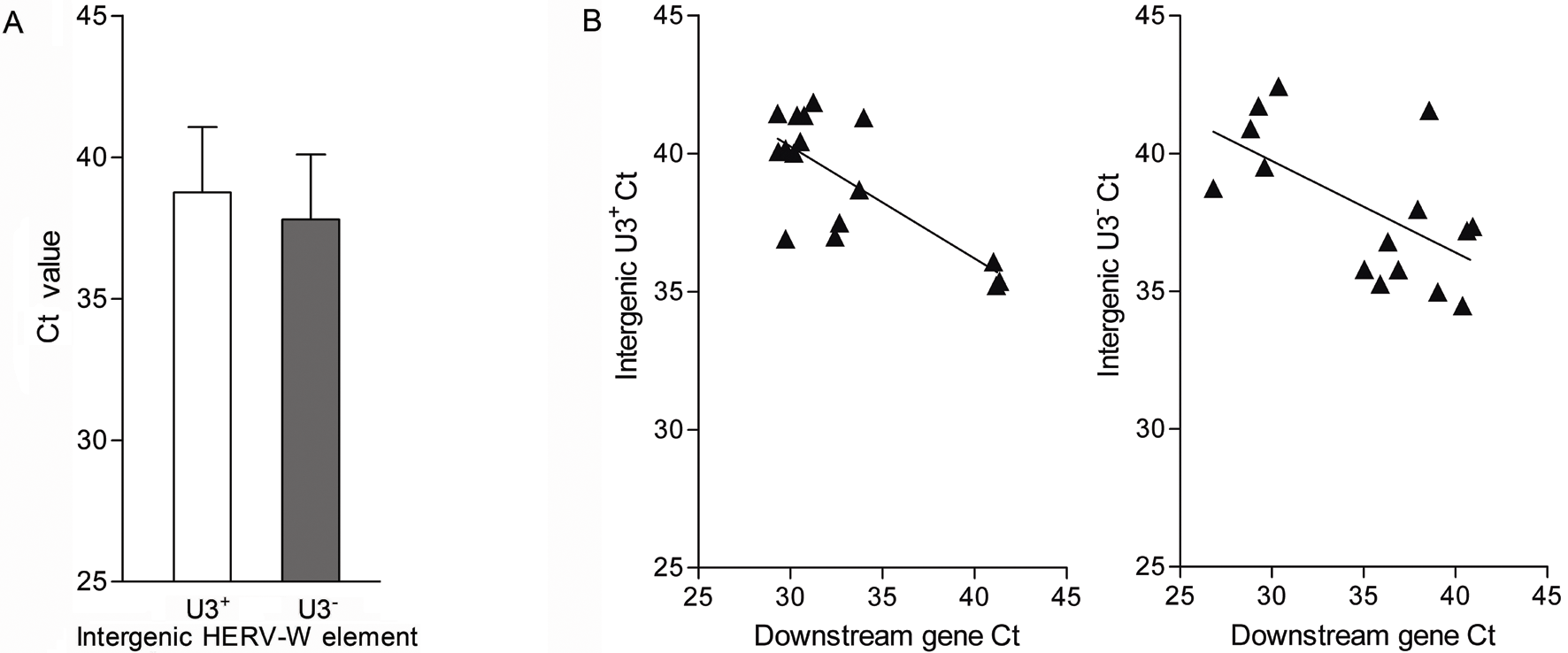

Supplement: Additional file 4 — Average levels of transcripts 3' of intergenic HERV-W elements and adjacent genes. Average levels of transcripts 3' of intergenic proviral elements (U3+, n = 5) and pseudoelements (U3-, n = 5) (A). Linear regression analyses of levels of transcripts 3' of intergenic U3+ and U3- elements and corresponding downstream genes (B). [file 1471-2164-12-22-S4.TIFF]
