# Supplementary material for: A systematic evaluation of expression of HERV-W elements; influence of genomic context, viral structure and orientation
Source: BMC Genomics. 2011 Jan 12;12:22. doi: 10.1186/1471-2164-12-22 (PMC3031232; doi:10.1186/1471-2164-12-22)
Supplement: Additional file 5 — Primers located inside HERV-W elements. This file describes the targets, product sizes, sequences and positions of primers used to link HERV-W elements with their corresponding assayed 3'-regions. [file 1471-2164-12-22-S5.PDF]

Table S1: Primers located inside HERV-W elements.

Target	Product (bp)	Polarity	Sequence	Position	Target	Product (bp)	Polarity	Sequence	Position
Intronic HERV-W proviral element					Intronic HERV-W pseudoelement				
CD72 intron 1	654	R	GAACCCCAAGTCAGAGAACACAA	chr9:35630372	*SLC16A10* intron 1	883	F	GATATAAACCCAGGCGTTCTAGC	chr6:111565868
TBX18 intron 7	1028	R	CGCCACCATCTTAGGAGCTCT	chr6:85478164	*FOXP2* intron 3	1270	R	TTTGGGTCCCCTCCATTTGTAT	chr7:113806428
ACOX3 intron 1	404	F	CACCACCCTGGGAGCAAAGA	chr4:8480366	*APG5* intron 6	1677	F	CACAGCGGGAAGGACAATGAT	chr6:106790254
ZNF678 intron 1	950	R	ATTGTTCCTGCGTGGGTAAG	chr1:225879069	*KIAA0423* intron7	962	R	AACGGCTACCCTCCTTGGGT	chr14:44558492
ANO3 intron 14	563	R	AACAAAAGTCTTGCCGCCATCT	chr11:26568631	*NRCAM* intron 2	724	F	CAATGGCTATGCTCTTCGGG	chr7:107772098
Intronic solitary LTR									
chrXq13.1	317	F	GAGCGGCCCACCACTATCTT	chrX:71350446					
chrXq26.2	954	R	CCATGGCTTCTGTTAGAACTGTAA	chrX:131381839					
chr6p22.1	724	F	ACCACCACATGACCCAAGATTC	chr6:29634583					
Intergenic HERV-W proviral element					Intergenic HERV-W pseudoelement				
pro chr12q14,1	1100	R	CCTGCCACCTTCTTGGTAGC	chr12:57531941	pse chr2q24,3	613	R	CGGGGCTTACAACTTAACTCACA	chr2:165222895
pro chr6p12,1	566	R	GCACCTTTAAACACGGGGC	chr6:52887671	pse chr3q23	1048	F	TGAGAGCACAGCGGGATATAAAC	chr3:143022933
pro chr7q33	1194	F	GGAACAAAGACCCACCCCATA	chr7:133928284	pse chr12p13.31	510	F	GCTGGGGAAGATGACTGTACTCA	chr12:7230122
pro chr10q21.2	976	R	GAACAAGGACTGACGCCCA	chr10:62463456	pse chr1q32.3	331	R	ATGGCGGGAGGGACAATGA	chr1:210095865
					pse chr6q15	667	R	CAGCAATGGCAATCCCCTTT	chr6:89180697
